# Mr. Dunning, on Vaccination

**Published:** 1802-01

**Authors:** Richard Dunning

**Affiliations:** Dock


### Medical and Phyfical Journal.

VOL. VII.] JANUARY, 1802. [NO. XXXV.

### To Dr. BATTY.

DEAR SIR,

IF you deem the following minute of a Cafe of premature determination to the Uterus, worth recording in your Journal, I will beg you to infert it, with the annexed Obfervations on Vaccination, in your next Number. I am, &c.

*Dock, Dec.* 5, 1801. RICHARD DUNNING.

A. B. always till within the laft three months, lived at Torpoint, a village on the banks of the Tamar, immediately oppofite this town. She was born a ftout healthy child, has continued almoft uninterruptingly to be fo, and was feven years old on the 11th of Auguft laft. More than two years and a half ago, fhe was obferved to be a good deal uneafy; her mother, from enquiries and obfervation, collected from her that fhe fuffered much pain in the lower part of her back and abdomen. The following morning, however, fhe was perfectly well, but the mother was much furprifed by the appearance of feveral patches of blood on her linen. About two months after, fimilar circumftances in every refpect again occurred, and have from that time to the prefent, (with the interruption of two periods only, in the intervals of which fhe was troubled with no inconfiderable quantity of leucorrhœa,) returned monthly with fo much regularity, that the mother can nearly tell the day of recurrence. The child has refided during the laft three months in Plymouth, in a clofe part of the town. I faw the mother yefterday with Dr. Remmett. A fecond month has now paffed without a return; but leucorrhœa has again taken place; the child has a tumid abdomen and begins to be indifpofed; the mother obferved to us, fhe believes fhe will remain fo, until *fhe becomes regular again.* It ftrikes me that the ftrength and character of the child's face are rather more that of juvenility than of childhood, and alfo, that the fullnefs and prominency of the breafts are greater than are common to fo early an age; no other marks of puberty attend. What her faculties or difpofitions are, I have not enquired. Recollecting it has been faid, that particular affociations or impreffions have been remarked through feveral fucceffions in fome families, I afked the mother and grandmother, who were prefent when I made thofe minutes, at what age menftruation began with them. Although they both told me, at a period rather earlier than ufual in this country, I do not, however, think the information enough remarkable to connect it at all with the child's cafe. I find a cafe among Tulpius's very valuable obfervations, ftrikingly fimilar. As this author may not be in every body's hands, and as the refult of the cafe fuggefts certainly a very ufeful practical hint, that we fhould not by any means attempt to fupprefs or even interrupt thefe fingular deviations of Nature, I have judged it worth while to tranfcribe the whole of it. Obfervationes Nicolai Tulpii, Capt. xxxvi. Lib. iii. Menftrua puellæ quadrimæ. Sed fupra fidem propè modum eft, filia cujufdam tabellarii, cui à quarto, in octavum ætatis annum periodicè ubi fluxiffent menftrua; accidit forte, ut illis fuppreffis, omnis ipforum fanguis converfus fit in capite producens illic primùm quidem dolorem dentis; fed mox fordidum, ac finuofum gingivarum ulcus; et tam pertinacem inferioris maxillæ cariem; ut non nifi tardiffime potuerit ulceris cavum carne iterum impleri, et convenienter cicatrice obduci, licet nihil non molitus fit nobifcum, vir fedulus, Daniel Arminius, Societati Indicæ à Medicinis.

---

